# bFGF rescues dysfunctional properties of adipose-derived stem cells from individuals with type 2 diabetes by modulating their miRNA profile

**DOI:** 10.1007/s00125-025-06533-0

**Published:** 2025-09-04

**Authors:** Anna Civit-Urgell, Esther Peña, Maria Teresa Bejar, Fabrizio Moscatiello, Gemma Vilahur, Lina Badimon, Gemma Arderiu

**Affiliations:** 1 Institut de Recerca Sant Pau (IR SANT PAU), Barcelona, Spain; 2 Facultat de Farmàcia i Ciències de l’Alimentació, Universitat de Barcelona (UB), Barcelona, Spain; 3 Centro de Investigación Biomédica en Red Cardiovascular (CIBER CV), Instituto de Salud Carlos III, Madrid, Spain; 4 Present Address: Cambridge Stem Cell Institute, University of Cambridge, Cambridge, UK; 5 Clínica Teknon, Grupo Quiron Salut, Barcelona, Spain; 6 School of Medicine, Universitat de Vic-UCC and Cardiovascular Research Foundation for Health Prevention and Innovation (FICSI), Barcelona, Spain

**Keywords:** Angiogenesis, ASCs, bFGF, Endothelial cells, MicroRNAs, Type 2 diabetes

## Abstract

**Aims/hypothesis:**

The aim of this study was to investigate whether basic fibroblast growth factor (bFGF) can restore the proliferation and migration capacities of adipose-derived stem cells (ASCs), which are impaired by type 2 diabetes, and improve vascular remodelling.

**Methods:**

ASCs obtained from individuals with or without diabetes were cultured with 10 ng/ml bFGF for 9 days. The ASCs were phenotypically characterised and functionally tested for proliferation capacity. Differentially expressed miRNAs before and after treatment were analysed using miRNA arrays. Crosstalk between ASCs and human vascular smooth muscle cells (HVSMCs) was assessed using wound healing, transwell migration and co-culture assays. Finally, a Matrigel plug assay in nude mice was used to evaluate the contribution of ASCs to neovessel formation.

**Results:**

bFGF treatment significantly enhanced the proliferation and migration of ASCs from individuals with type 2 diabetes (T2DM ASCs), and altered the expression of miRNAs associated with ASC proliferation. ASCs promoted HVSMC migration and, when co-cultured, facilitated tube-like structure formation. In vivo Matrigel plug assays revealed that bFGF treatment enhanced neovessel formation. Although both non-T2DM ASCs (ASCs from individuals without type 2 diabetes) and untreated T2DM ASCs stimulated angiogenesis, bFGF-treated subcutaneous and visceral T2DM ASCs promoted even greater neovessel formation. Additionally, bFGF treatment modulated the expression of multiple angiogenesis-related miRNAs in ASCs.

**Conclusions/interpretation:**

Preconditioning T2DM ASCs with bFGF alters their miRNA profile, enhancing cell proliferation and their vascular remodelling potential. This strategy could improve the therapeutic utility of T2DM ASCs.

**Graphical Abstract:**

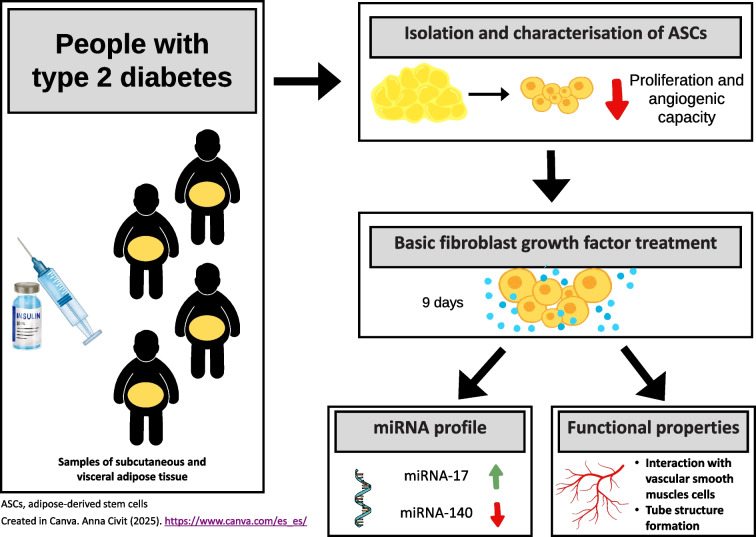


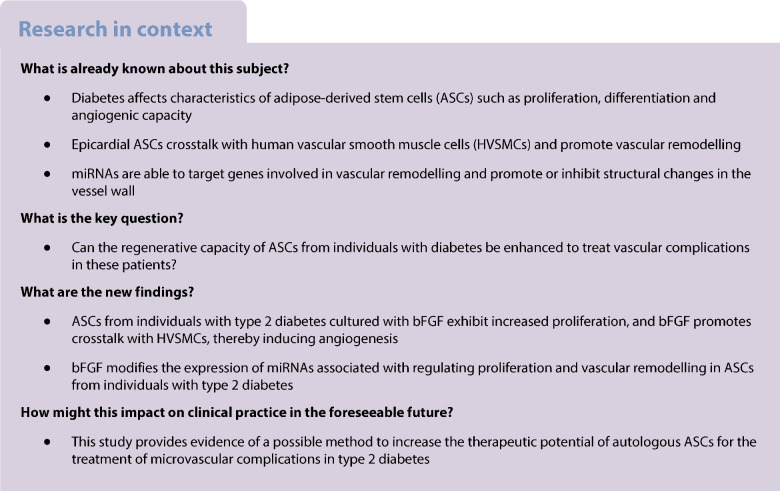


## Introduction

Diabetes mellitus is a complex syndrome of chronic hyperglycaemia encompassing several diseases that may lead to complications and even death [1, 2]. Vascular complications of diabetes are a major source of morbidity and mortality. Diabetes triggers vascular remodelling through various mediators and pathways, resulting in substantial alterations to the structure and function of all layers of the arterial wall. This remodelling leads to endothelial dysfunction, increased extracellular matrix deposition, vascular smooth muscle cell proliferation, and calcification, ultimately reducing vascular compliance and impairing tissue perfusion.

Vessel integrity was previously believed to be solely maintained by resident cells. However, recent evidence has shown that progenitor cells play a crucial role in re-endothelialisation and arterial remodelling following vascular injury [3]. Recent studies focusing on stem cell isolation and differentiation methodologies have highlighted adipose-derived stem cells (ASCs) as a promising source of stem cells for enhancing vascular remodelling. The capacity of ASCs to promote blood vessel formation is primarily linked to their paracrine effects [4, 5], as well as their ability to differentiate into endothelial cells or integrate into vascular structures [6, 7], which may help stabilise these structures during network formation.

ASCs are adult mesenchymal stem cells that are derived from adipose tissue. These cells contribute to the turnover of mature adipocytes and exhibit pro-angiogenic, anti-apoptotic and immunoregulatory properties [8–11] that make them excellent candidates for use in cell therapy. However, several studies involving human-derived cells and animal models have demonstrated that ASCs from individuals with diabetes have more limited therapeutic potential [12–14]. Ferrer-Lorente et al described the detrimental impact of diabetes on the therapeutic properties of ASCs, suggesting that the efficiency of autologous ASC transplantation in individuals with diabetes will be limited [14]. Type 2 diabetes increases ASC stemness, apoptosis and senescence, while reducing their proliferation potential and immunosuppressive functions [15]. Given the significant influence of the cellular environment on ASCs, in vitro research has focused on manipulating the culture conditions to guide differentiation along specific lineages. In this context, basic fibroblast growth factor (bFGF) has been found to induce angiogenesis, wound healing and vascular remodelling [16, 17]. In addition, miRNAs are potential therapeutic targets due to their gene regulatory function. miRNAs are tissue- and cell-type-specific and target genes involved in angiogenesis, inflammation and senescence pathways [18], as well as having regulatory functions in the proliferation and differentiation of ASCs [7, 19–22].

In this context, we hypothesised that bFGF could modulate the gene expression profile of subcutaneous and visceral ASCs from individuals with obesity and type 2 diabetes to restore the stem cell function impaired by diabetes and promote microvascular vessel formation, stabilisation and maturation, thereby ameliorating vascular dysfunction associated with excess fat accumulation. We also identified specific miRNAs as potential candidates involved in restoring the impaired properties of ASCs induced by type 2 diabetes.

## Methods

### Participants and samples

Adipose tissue from subcutaneous and visceral fat depots was obtained via surgical resection from individuals with type 2 diabetes (BMI >40 kg/m^2^; *n*=4) who underwent bypass gastric surgery. Additionally, we collected adipose tissue from non-diabetic individuals with a BMI ≤25 kg/m^2^ (*n*=4) who underwent abdominal lipectomy. Samples were kept as a biological repository, which was approved by the Hospital de Sant Pau Ethical Committee (collection 01/2020). Sex was self-reported; race or ethnicity data were not collected. The tissue samples were anonymised when passed from the surgical room to the laboratory technician in charge of taking the tissue to the cell culture facilities. Another code was provided when entering the tissue culture facility. Therefore, no possible link from the tissue to the donor could be established. Isolated cells in passage 1 were kept frozen until used. Information on a minimal set of clinical parameters was associated with the code. Details regarding the individuals from whom the cells used in this study were obtained are presented in Table 1. This table also includes clinical and demographic data for the individuals in the GSE245003 dataset.

**Table 1:** Source of the stem cells used in this study

Individuals	Tissue sample	Surgery	Age (years)	Sex	BMI (kg/m^2^)	Weight (kg)	Glucose (mmol/l)	HbA_1c_ (mmol/mol)	HbA_1c_ (%)
Non-diabetic (*n*=4)	Subcutaneous	Abdominal lipectomy	39.0 ± 12.9	Three women, one man	23.35 ± 4.47	68 ± 17.70			
Non-diabetic obese (*n*=4)^a^	Subcutaneous and visceral	Gastric bypass and cholecystectomy	47.5 ± 9.6	Three women, one man	39.15 ± 8.89	118.50 ± 27.16			
Diabetic (*n*=4)	Subcutaneous and visceral	Gastric bypass and cholecystectomy	52 ± 8.3	Two women, two men	45.86 ± 4.17	122.4 ± 15.75	10.61 ± 1.50	68.85 ± 18.30	8.60 ± 1.67

Values are means ± SD

^a^The group of non-diabetic individuals undergoing gastric bypass and cholecystectomy refers to data from GSE245003 (see Methods)

### Isolation, cell surface marker characterisation and culture of ASCs

Isolation of primary human ASCs was performed as previously described [13]. Isolated stromal vascular fraction cells were counted and either analysed by flow cytometry or seeded into T-25 culture flasks (TPP, Reactiva). After 24 h, non-adherent cells were removed and the medium replaced. Cells were expanded in ASC culture medium (DMEM with low glucose [Thermo Fisher Scientific] supplemented with 10% FBS [Biological Industries, Kibbutz Beit-Haemek, Israel], 100 U/ml penicillin and 0.1 mg/ml streptomycin [Invitrogen]) between passages 0 and 4, and cultured at 37°C, 5% CO_2_ and 95% air humidity to a sub-confluent state. The identity of ASCs was defined by cell surface antigen phenotype as described by Oñate et al [12]. Expression of CD105, CD90, CD29, CD44, CD45 and CD34 markers was determined by flow cytometry.

### HVSMC isolation and culture

Primary cultures of human vascular smooth muscle cells (HVSMCs) were obtained from the ascending aortas of explanted hearts of individuals undergoing cardiac transplantation at the Hospital de la Santa Creu i de Sant Pau. Tissue was obtained with the individuals’ informed consent (*n*=6) and the protocol was approved by the research ethics committee at the study centre and was performed in accordance with the Declaration of Helsinki (S4/2014). HVSMCs were obtained by a modification of the explant technique described previously [23] and cultured in M199 medium (Gibco) containing 20% FBS, 100 U/ml penicillin, 0.1 mg/ml streptomycin and 2 mmol l-glutamine (Gibco). The medium was replaced every 2 days.

### ASC culture with bFGF

ASCs were cultured in ASC medium supplemented with 10 ng/ml bFGF (233-FB, R&D Systems, Minneapolis, MN, USA) for 9 days [24].

### MTS viability/proliferation assay

Cell proliferation was determined using a 3-(4,5-dimethylthiazol-2-yl)-5-(3-carboxymethoxyphenyl)-2-(4-sulfophenyl)-2H-tetrazolium (MTS) cell proliferation assay kit (colorimetric) (ab197010) (Abcam, Cambridge, UK) according to the manufacturer’s instructions. For this assay, 1 × 10^3^ cells were seeded in quadruplicate into a 96-well microtitre plate (Corning Costar, Corning, NY, USA). ASCs were cultured for 24 h with ASC medium or ASC medium supplemented 10 ng/ml bFGF. The absorbance was then quantified using a Spectramax 250 spectrophotometer [Molecular Devices, San Jose, CA, USA], and analysed using SoftMax software version 2.0.16 (Molecular Devices). Formazan production as measured using the spectrophotometer was directly related to the number of cells alive in the culture.

### Wound repair assay

Wound repair assays were performed using Cultures-Insert Petri dishes (Ibidi, Martinsried, Germany). Confluent monolayers of ASCs were seeded on one side of the insert and HVSMCs were seeded on the other side. Wound repair was assessed using either untreated ASCs or ASCs previously treated for 9 days with bFGF. Images were acquired after 10 h under a 10× objective, and further digitalised and processed using a Leica DMIRE2 microscope attached to a video SPOT Leica-DFC350FX camera. Wound areas were analysed by using ImageJ software (National Institutes of Health, USA).

### Transwell migration assay

The chemotaxis assay was performed using transwell chambers. The experiments were performed six times using cells from six different patients; cells from each patient were used for the seven types of conditions. Cells were plated into modified Boyden chambers (Corning Costar) as described previously [25]. Briefly, 6.5 mm transwell chambers with 8 μm pores were coated with 10 μg/ml type I collagen (Sigma) for 2 h at 37°C. A total of 5 × 10^4^ HVSMCs per well were seeded in the upper chamber, and the lower chamber was filled with ASC medium (described above), ASC medium supplemented with 10 ng/ml bFGF or with 1.5 × 10^4^ ASCs/well (untreated or previously treated for 9 days with 10 ng/ml bFGF). After 4 h at 37°C, non-migratory cells in the upper chamber were removed using a cotton swab, and cells that had migrated to the bottom of the membrane were fixed and stained using Diff-Quick (VWR Scientific Products). The total number of migrated cells was determined by counting five fields in each well per experimental condition using a phase-contrast microscope with a 20× objective.

### Matrigel assay: capillary-like structure formation

Three-dimensional cultures were prepared on basement membrane (BD Matrigel, BD Biosciences) as described previously [26]. The co-culture system comprised HVSMCs and subcutaneous and visceral ASCs from individuals with or without type 2 diabetes (T2DM ASCs and non-T2DM ASCs) that were untreated or previously treated for 9 days with bFGF. To discriminate between each cell type, cells were labelled with two different living-cell fluorescent membrane dyes, PKH67 for ASCs and PKH26 for HVSMCs (Sigma-Aldrich). Cell movements were monitored by time-lapse video microscopy at 15 min intervals. Cells were viewed using a Leica PL APO 20×/0.7 multi-immersion CS objective. Images were acquired, digitalised and processed using Leica TCS-AOBS software.

### In vivo Matrigel plug angiogenesis assay

The Matrigel implantation assay was performed as previously described [6]. Animal care and all experimental procedures were performed in strict accordance with the approved protocols and animal welfare regulations of the Institutional Animal Care and Use Committee (CEEA) and authorised by the Animal Experimental Committee of the local government (#ICCC041) in accordance with Spanish law (RD 53/2013) and European Directive 2010/63/EU. Co-cultures of ASCs from individuals without type 2 diabetes (non-T2DM ASCs), subcutaneous and visceral T2DM ASCs (untreated or previously treated for 9 days with bFGF) (2 × 10^6^) with HVSMCs (2 × 10^6^) prepared in 150 μl Matrigel medium were injected subcutaneously into the flank region of nude mice (R/SOPF BALB/C NU/NU CBy*.*Cg-*Foxn1*^nu/j^, Charles River Laboratories [Wilmington, MA], https://www.jax.org/strain/000711). All mice (*n*=6) were euthanised at 7 days post-injection, and the skin of the mouse was pulled back to expose the Matrigel plug, which remained intact. The skin underlying the plugs was visualised macroscopically using a Leica AF 6000LX stereo microscope with DFC digital camera (8-bit resolution, objectives 1.0 × 0.03/1.0 × 0.09). Matrigel implants were isolated from the surrounding tissues, photographed and subjected to content analysis.

### Haemoglobin assessment

Haemoglobin content was analysed using the cyanohaemoglobin method (Drabkin’s method) modified by us for tissue use as previously described [27].

### miRNA extraction and quantification from ASC cultures

miRNA and total RNA were isolated using a mirVana miRNA isolation kit with phenol (Life Technologies), according to the manufacturer’s instructions. cDNA was synthesised using a TaqMan Advanced miRNA cDNA synthesis kit (Life Technologies). An ND-1000 ultraviolet spectrophotometer (Nanodrop) was used to quantify the extracted miRNA.

### miRNA or anti-miRNA transfection

miRNA mimic and/or miRNA inhibitors (AMBION by Life Technologies) were transfected into ASCs in six-well plates at the following concentrations: miR-17-5p mimic (MC12412), 50 nmol/l; miR-140-5p inhibitor (MH10205), 200 nmol/l; mirVana negative control mimic/inhibitor, 50 nmol/l. Cells were transfected using Lipofectamine RNAiMAX reagent (Life Technologies), according to the manufacturer’s protocol.

### qRT-PCR analysis of miRNA expression

TaqMan real-time qRT-PCR was performed using an ABIPRISM 7900HT Fast real-time PCR system (Applied Biosystems), and the analysis was performed using SDS software version 2.4 (Applied Biosystems). miRNA levels were quantified using hsa-miRNA-17-5p (478447_mir) and hsa-miRNA-140-5p (477909_mir) and the internal normalised control hsa-miRNA-186-5p (477940_mir) (Applied Biosystems). The results were normalised to hsa-miRNA-186-5p.

#### Data source and pre-processing

A 500 ng aliquot from each extracted RNA sample was analysed using GeneChip miRNA 4.0 arrays (Thermo Fisher Scientific). Samples were processed according to the manufacturer’s instructions with minimal modifications [28], using a FlashTag biotin HSR RNA labelling kit (Thermo Fisher Scientific) and the GeneChip Scanner 3000 7G system (including the GeneChip Scanner 3000 7G, Fluidics Station 450 and Hybridisation Oven 645).

The miRNA profile was downloaded from the Gene Expression Omnibus (GEO; https://www.ncbi.nlm.nih.gov/geo/). We further screened the miRNAs according to our criteria, using *p*<0.05 and |log_2_FC|>1 or |log_2_FC|>3 as thresholds (FC, fold change).

#### Integrated target prediction

Bioinformatic prediction for potential target genes was performed using the miRNA data integration protocol (mirDIP) available at https://ophid.utoronto.ca.

#### Pathway and functional enrichment analysis

Enrichment analysis of the targeted-associated genes was evaluated using the web-based software ShinyGO version 0.82 (available at http://bioinformatics.sdstate.edu), the Database for Annotation, Visualisation and Integrated Discovery bioinformatics resources (available at https://davidbioinformatics.nih.gov/) and the PANTHER classification system (available at https://pantherdb.org). Values for *p*<0.05 were considered significant. Gene ontology terms and Kyoto Encyclopedia of Genes and Genomes (KEGG) pathways were sorted by average and rank.

#### Construction of miRNA regulatory network and visualisation analysis

Cytoscape version 3.9.0 (https://cytoscape.org) was used to build protein–protein interaction networks according to STRING database interaction data (https://string-db.org/). The confidence cut-off was set to 0.8, and the maximum number of additional interactions was limited to 30. In order to identify protein–protein interaction-enriched clusters, a community cluster strategy was applied to the main connected components of each network.

#### Statistical analysis

Statistical calculations were performed on at least three independent experiments. The Shapiro–Wilk test was applied to determine normal distribution of the data, and intergroup comparisons were performed using a two-tailed unpaired Student’s *t* test or Wilcoxon matched-pairs signed rank test. Grouped analyses were performed by non-parametric Kruskal–Wallis or parametric one-way ANOVA/mixed-effects analysis with Tukey’s multicomparison test. The statistical software package GraphPad Prism version 10.1.1 (GraphPad Software, San Diego, CA, USA) was used for statistical analyses. The results are expressed as means ± SD, and the number of experiments is indicated. Differences were considered statistically significant when *p*<0.05.

## Results

### Effects of bFGF on the proliferation capacity of subcutaneous and visceral T2DM ASCs

**Fig. 1:**
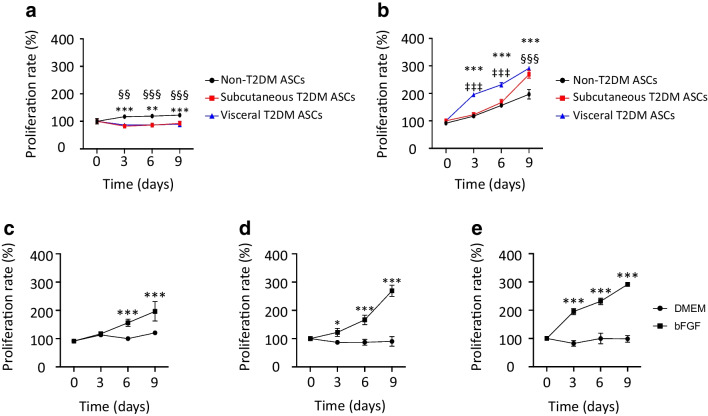
Effects of bFGF on the proliferation potential of T2DM ASCs. (**a**) MTS cell proliferation assay for non-T2DM ASCs, subcutaneous T2DM ASCs and visceral T2DM ASCs cultured with DMEM. Data are means ± SD (*n*=4). Statistical analysis was performed by one-way ANOVA followed by Tukey test: **p<0.01 and ***p<0.001, non-T2DM ASCs vs subcutaneous T2DM ASCs; ^§§^*p*<0.01 and ^§§§^*p*<0.001, non-T2DM ASCs vs visceral T2DM ASCs. (**b**) MTS cell proliferation assay for non-T2DM ASCs, subcutaneous T2DM ASCs and visceral T2DM ASCs cultured with DMEM + bFGF (10 ng/ml). Data are means ± SD (*n*=4). Statistical analysis was performed by one-way ANOVA followed by Tukey test: ***p<0.001, non-obese vs visceral T2DM ASCs; ^‡‡‡^*p*<0.001, subcutaneous T2DM ASCs vs visceral T2DM ASCs; ^§§§^*p*<0.001, non-obese vs subcutaneous T2DM ASCs. (**c**) MTS cell proliferation assay for non-T2DM ASCs cultured with DMEM or DMEM + bFGF (10 ng/ml). Data are means ± SD (*n*=4). Statistical analysis was performed by two-way ANOVA followed by Tukey test: ***p<0.001, DMEM vs DMEM + bFGF. (**d**) MTS cell proliferation assay for subcutaneous T2DM ASCs cultured with DMEM or DMEM + bFGF (10 ng/ml). Data are means ± SD (*n*=4). Statistical analysis was performed by two-way ANOVA followed by Tukey test: *p<0.05 and ***p<0.001, DMEM vs DMEM + bFGF. (**e**) MTS cell proliferation assay for visceral T2DM ASCs cultured with DMEM or DMEM + bFGF (10 ng/ml). Data are means ± SD (*n*=4). Statistical analysis was performed by two-way ANOVA followed by Tukey test: ***p<0.001 DMEM vs DMEM + bFGF

ASCs were isolated from subcutaneous and visceral adipose tissue from individuals with type 2 diabetes and characterised by flow cytometry. As it has been demonstrated that type 2 diabetes causes ASC dysfunction, we compared the proliferation rates of ASCs from individuals with and without type 2 diabetes. The MTS assay showed a reduction in the proliferation capacity of subcutaneous and visceral T2DM ASCs in comparison with non-T2DM ASCs even after only 3 days of culture (*p*<0.001). The significance increased after 9 days of culture for both subcutaneous and visceral T2DM ASCs (*p*<0.001) (Fig. 1a). We further evaluated the ability of bFGF to ameliorate the impaired functions of ASCs. bFGF significantly enhanced growth rates in subcutaneous and visceral fat depots, with the greatest effect observed in visceral T2DM ASCs (Fig. 1b). In both subcutaneous and visceral T2DM ASCs, this capacity was improved on day 3 (*p*<0.05 and *p*<0.001, respectively), although visceral T2DM ASCs proliferated faster than subcutaneous T2DM ASCs. In non-T2DM ASCs, the proliferation capacity was improved on day 6 (*p*<0.001) (Fig. 1c–e).

### bFGF modifies the miRNAs involved in proliferation of subcutaneous and visceral T2DM ASCs

**Fig. 2:**
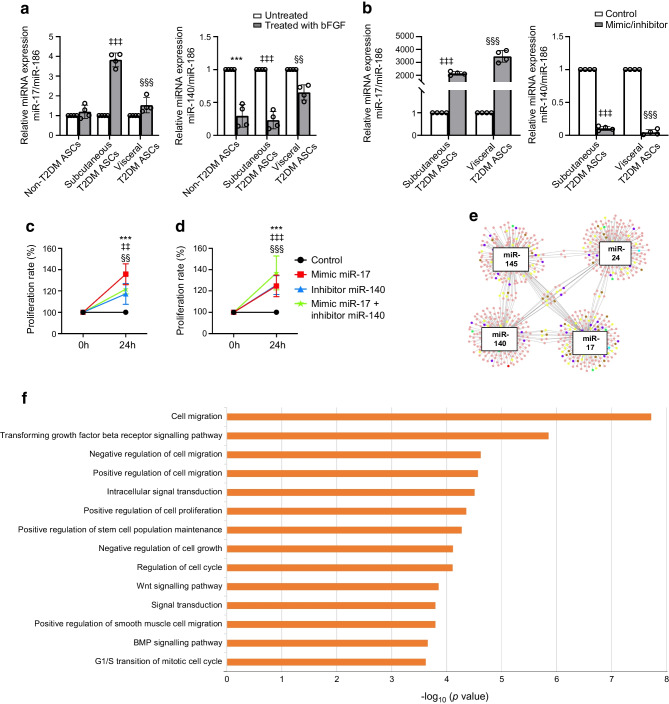
Effects of miR-19 and miR-140 on ASC proliferation capacity. (**a**) Relative miR-17 and miR-140 expression in untreated ASCs and after treatment with bFGF for 9 days. Data are means ± SD (*n*=4). Statistical analysis was performed by two-way ANOVA followed by Tukey test: ***p<0.001, untreated vs non-T2DM ASCs; ^‡‡‡^*p*<0.001, untreated vs subcutaneous T2DM ASCs; ^§§^*p*<0.01 and ^§§§^*p*<0.001, untreated vs visceral T2DM ASCs. (**b**) Relative miR-17 and miR-140 expression after treatment of T2DM ASCs with negative control (mimic/inhibitor negative control) or miR-17 mimic and miR-140 inhibitor. Data are means ± SD (*n*=4). Statistical analysis was performed by one-way ANOVA followed by Tukey test: ^‡‡‡^*p*<0.001, negative control vs transfected subcutaneous T2DM ASCs; ^§§§^*p*<0.001, negative control vs transfected visceral T2DM ASCs. (**c**) MTS cell proliferation assay for subcutaneous T2DM ASCs after transfection with miR-17 mimic, miR-140 inhibitor or co-transfection with miR-17 mimic and miR-140 inhibitor. Data are means ± SD (*n*=4). Statistical analysis was performed by two-way ANOVA followed by Tukey test: ***p<0.001, 0 h vs 24 h miR-17 mimic transfection; ^‡‡^*p*<0.01, 0 h vs 24 h miR-140 inhibitor transfection; ^§§^*p*<0.01, 0 h vs 24 h miR-17 mimic and miR-140 inhibitor co-transfection. (**d**) MTS cell proliferation assay for visceral T2DM ASCs after transfection with miR-17 mimic, miR-140 inhibitor or co-transfection with miR-17 mimic and miR-140 inhibitor. Data are means ± SD (*n*=4). Statistical analysis was performed by two-way ANOVA followed by Tukey test: ***p<0.001, 0 h vs 24 h miR-17 mimic transfection; ^‡‡‡^*p*<0.001, 0 h vs 24 h miR-140 inhibitor transfection; ^§§§^*p*<0.001, 0 h vs 24 h miR-17 mimic + miR-140 inhibitor co-transfection. (**e**) miRNAs involved in T2DM ASC proliferation. Yellow dots represent genes involved in cell migration, purple dots represent genes involved in proliferation, brown dots represent genes involved in cell cycle, red dots represent genes involved in cell migration, proliferation and the cell cycle, green dots represent genes involved in cell migration and proliferation, and blue dots represent genes involved in cell migration and the cell cycle. The network was visualised using Cytoscape version 3.9.0. (**f**) Enriched biological processes of those miRNAs involved in T2DM ASC proliferation. BMP, bone morphogenic protein

It has been reported that diabetes mellitus impairs ASC proliferation capacity by overexpression of several miRNAs [29]. We investigated whether bFGF modulated the transcriptomic profile of ASCs from individuals with type 2 diabetes, modifying those miRNAs involved in cell proliferation, senescence and apoptosis. In our array data (*n*=4), subcutaneous T2DM ASCs treated with bFGF were characterised by suppressed expression of miR-24, miR-145 and miR-140 and increased expression of miR-17 compared with untreated cells. However, only miR-140 expression was inhibited and miR-17 expression was increased in visceral T2DM ASCs after bFGF treatment (Table 2). Given these findings, expression of miR-17 and miR-140 was validated in our samples by qRT-PCR analysis. Expression of miR-17-5p showed an increase (*p*<0.001) in both subcutaneous and visceral T2DM ASCs, whereas expression of miR-140-5p was downregulated after treating subcutaneous and visceral T2DM ASCs (*p*<0.001 and *p*<0.01, respectively) with bFGF for 9 days (Fig. 2a). Moreover, we also analysed the expression levels of these miRNAs in untreated non-T2DM ASCs and after bFGF treatment, but only observed significant differences in the expression of miR-140 (Fig. 2a).

**Table 2:** Expression levels of the miRNAs involved in ASCs proliferation capacity

miRNAs	Non-T2DM ASCs	Subcutaneous T2DM ASCs	Visceral T2DM ASCs
miR-17-5p	3.84	4.67*	1.56*
miR-24-3p	−2.20*	−1.77*	−0.78
miR-140-5p	−1.51	−12.16*	−4.22*
miR-145-5p	−9.55*	−7.65*	−1.08

Values are log_2_ FC

The miRNA expression was compared between untreated ASCs and ASCs previously treated for 9 days with bFGF. Asterisks indicate *p*<0.05 for comparison of treated vs untreated ASCs

To demonstrate the role of these miRNAs in the proliferation capacity of T2DM ASCs, we performed miRNA functional assays followed by MTS cell proliferation assays. As shown in Fig. 2b, after mimic transfection, the expression of miR-17 was significantly upregulated in all T2DM ASCs (*p*<0.001). This upregulation resulted in increased proliferation capacity (*p*<0.001) after 24 h of transfection in both subcutaneous (Fig. 2c) and visceral (Fig. 2d) T2DM HVSMCs. Similarly, transfection of the miR-140 inhibitor resulted in decreased miRNA expression (*p*<0.001 for both subcutaneous and visceral T2DM ASCs) (Fig. 2b) and increased proliferation capacity (*p*<0.01 and *p*<0.001) after 24 h of transfection in subcutaneous (Fig. 2c) and visceral T2DM ASCs, respectively (Fig. 2d). In addition, co-transfection of mimic and inhibitor showed increased growth rates compared with mimic and inhibitor controls (Fig. 2c, d). Together, these results suggest that miR-140 inhibition and miR-17 upregulation regulate the proliferative capacity of T2DM ASCs following bFGF treatment.

We used the high confidence option in mirDIP to target genes that were related to miRNAs involved in proliferation. Collectively, miR-24, miR-145, miR-140 and miR-17 were predicted to target 804 genes with high stringency that were above the bottom third in confidence in at least ten databases (Fig. 2e). The majority of the genes substantially contributed to specific processes related to cell migration (71 genes) or cell proliferation and stem cell population maintenance (41 genes) (Fig. 2f). The over-represented pathways included the TGFβ signalling pathway (14 genes), the integrin signalling pathway (23 genes) and the Wnt signalling pathway (23 genes). These results indicate that bFGF can modulate the miRNA profile in ASCs, enhancing their proliferative capacity.

### miR-17, miR-20a, miR-222, miR-23b and miR-27b regulation in ASCs is independent of BMI status or disease

Several angiogenesis-related miRNAs have been shown to be abundantly expressed in human ASCs [30, 31]. Here, we aimed to determine whether the expression of these miRNAs was altered following bFGF treatment, using bioinformatic analysis. Of the 37 reported angiogenic-related miRNAs, we identified 19 miRNAs in our samples, including miR-17. However, even though 19 were present in subcutaneous T2DM ASCs after bFGF treatment, only four were significantly regulated in visceral T2DM ASCs after bFGF treatment (Table 3), thus only four angiogenesis-related miRNAs are regulated by bFGF in both subcutaneous and visceral ASCs.

**Table 3:** Fold change (FC) in angiogenesis-related miRNAs that are inhibited in human T2DM ASCs after bFGF culture

miRNA	FC subcutaneous T2DM ASCs	FC visceral T2DM ASCs
hsa-let-7d	0.31	
hsa-let-7e	0.01	
hsa-let-7f	0.14	
hsa-let-7g	0.37	
hsa-miRNA-10a	0.02	
hsa-miRNA-10b		
-3p	0.0004	
-5p	0.03	
hsa-miRNA-125a-5p	0.18	
hsa-miRNA-132	0.41	
hsa-miRNA-143		
-3p	0.003	
-5p	0.002	0.06
hsa-miRNA-145	0.005	
hsa-miRNA-21	0.05	
hsa-miRNA-23b		
-3p	0.1	
-5p	0.01	0.008
hsa-miRNA-24		
-3p	0.29	
-5p	0.02	
hsa-miRNA-26a		
-3p	0.43	
-5p	0.44	
hsa-miRNA-26b	0.15	
hsa-miRNA-27a	0.11	
hsa-miRNA-27b		
-3p	0.02	0.05
-5p	0.002	0.005
hsa-miRNA-424		
-3p	0.007	
-5p	0.0002	
hsa-miRNA-503		
-3p	0.0008	
-5p	0.003	

Values represent fold change ratio relative to pre-treatment; only miRNAs with *p*<0.05 and absolute fold-change ≥1 are shown

To obtain further insight into whether regulation of these miRNAs is condition-specific (type 2 diabetes and/or obesity) or tissue-specific (subcutaneous or visceral), we compared their expression levels with those in non-T2DM ASCs as well as with the levels of miRNA expression in subcutaneous/visceral ASCs from obese donors without type 2 diabetes extracted from GSE245003 (Table 4). The analysis revealed that several of the miRNAs identified as elevated in ASCs were inhibited by bFGF under at least one condition and/or in at least one tissue. We found that expression of miR-23b and miR-27b was downregulated in all samples, and that miR-17, miR-20a and miR-222 were upregulated in all samples. Other miRNAs were regulated by bFGF in subcutaneous ASCs independently of the BMI status of the individual. miR-21 was downregulated in subcutaneous ASCs from individuals with obesity with or without type 2 diabetes. However, the role of bFGF on the regulation of 13 miRNAs was independent of the presence or absence of type 2 diabetes. Six miRNAs were only inhibited in subcutaneous T2DM ASCs, whereas only one miRNA was downregulated in subcutaneous ASCs from individuals with obesity. Taken together, these results indicate that miR-17, miR-20a and miR-222 were upregulated in all tissue samples regardless of their origin and that and miR-23b and miR-27b were inhibited in all tissue samples.

**Table 4:** Expression of angiogenesis-related miRNAs in human subcutaneous and visceral ASCs from individuals without obesity, with obesity or with type 2 diabetes

	Subcutaneous ASCs			Visceral ASCs	
miRNA	Non-obese	Obese	Type 2 diabetes	Obese	Type 2 diabetes
hsa-let-7b		−			
hsa-let-7c	**−**				
hsa-let-7d	**−**	**−**	**−**	**−**	
hsa-let-7e	**−**	**−**	**−**		
hsa-let-7f			**−**		
hsa-let-7g			**−**		
hsa-miR-10a			**−**		
hsa-miR-10b	**−**	**−**	**−**		
hsa-miR-125a-5p	**−**	**−**	**−**		
hsa-miR-132			**−**		
hsa-miR-143	**−**	**−**	**−**		**−**
hsa-miR-145	**−**	**−**	**−**		**−**
hsa-miR-155	**+**	**+**		**+**	
hsa-miR-15b	**+**	**+**	**+**		
hsa-miR-16	**+**	**+**	**+**		**+**
hsa-miR-17	**+**	**+**	**+**	**+**	**+**
hsa-miR-199a-3p				**+**	**+**
hsa-miR-199a-5p				**+**	**+**
hsa-miR-20a	**+**	**+**	**+**	**+**	**+**
hsa-miR-21		**−**	**−**		
hsa-miR-214				**+**	**+**
hsa-miR-221	**+**	**+**	**+**	**+**	
hsa-miR-222	**+**	**+**	**+**	**+**	**+**
hsa-miR-23a					**+**
hsa-miR-23b	**−**	**−**	**−**	**−**	**−**
hsa-miR-24	**−**	**−**	**−**		
hsa-miR-25	**+**	**+**	**+**		
hsa-miR-26a			**−**		
hsa-miR-26b	**+**		**−**		
hsa-miR-27a	**−**	**−**	**−**		
hsa-miR-27b	**−**	**−**	**−**	**−**	**−**
hsa-miR-29a	**+**	**+**			
hsa-miR-424	**−**	**−**	**−**		
hsa-miR-503	**−**	**−**	**−**		
hsa-miR-92a	**+**	**+**	**+**	**+**	

### Effects of bFGF on T2DM ASC crosstalk with HVSMCs and angiogenic potential

**Fig. 3:**
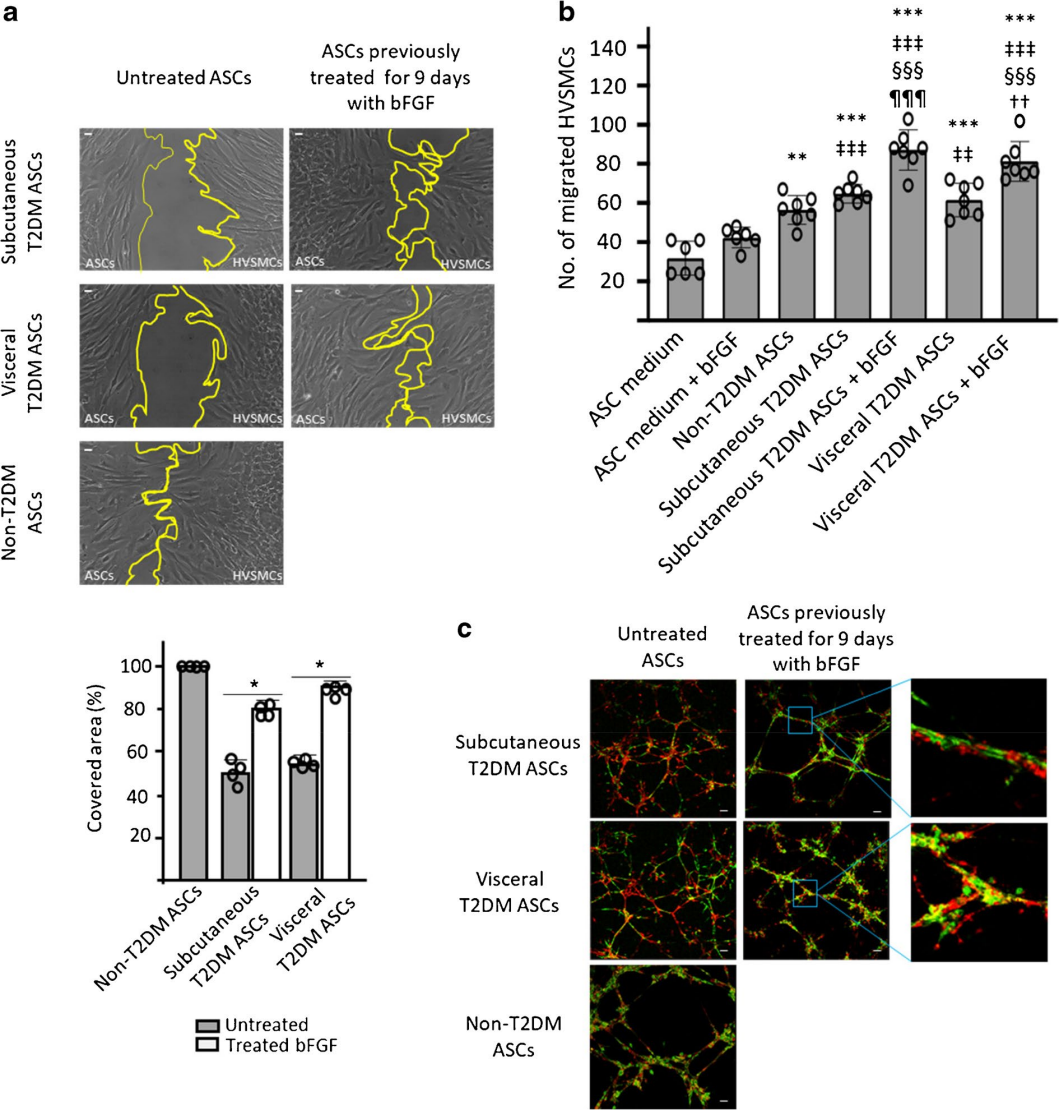
Effects of bFGF on the chemoattractant capacity of T2DM ASCs. (**a**) Phase-contrast micrographs showing wound repair in untreated ASC co-cultures or those previously treated for 9 days with bFGF (left of each image) and HVSMCs (right of each image). The accompanying graph shows the migrated cell surface as a percentage of the control (non-T2DM ASCs) at 10 h*.* Data are means ± SD (*n*=4). Statistical analysis was performed by one-way ANOVA followed by Tukey test: *p<0.05, untreated vs treated bFGF. Scale bar, 100 nm. (**b**) Chemotaxis assays were carried out using transwell culture chambers. The following combinations were added to the lower wells for 9 days of culture: ASC medium, ASC medium + bFGF (10 ng/ml), non-T2DM ASCs, subcutaneous T2DM ASCs, subcutaneous T2DM ASCs + bFGF (10 ng/ml), visceral T2DM ASCs or visceral T2DM ASCs + bFGF (10 ng/ml). HVSMCs (5 × 10^4^ cells) were seeded into the upper wells, and migrated cells were counted after 4 h. Values are means ± SD (*n*=6). Statistical analysis was performed by one-way ANOVA followed by Tukey test: **p<0.01 and ***p<0.001, vs ASC medium; ^‡‡^*p*<0.01 and ^‡‡‡^*p*<0.001, vs ASC medium + bFGF; ^§§§^*p*<0.001 vs non-T2DM ASCs; ^¶¶¶^*p*<0.001 vs subcutaneous T2DM ASCs; ††*p*<0.01 vs visceral T2DM ASCs. (**c**) Tubular organised structures of ASCs in combination with HVSMCs. Images show T2DM ASCs previously treated for 9 days with bFGF (red) and HVSMCs (green) after 18 h in a Matrigel co-culture system. Scale bar, 50 µm

It has been shown that type 2 diabetes attenuates crucial functions of ASCs, such as proliferation, viability and secretory activity [29]. We evaluated whether ASCs contribute to vascular remodelling through their migration capacity and paracrine effect on other vascular cells such as HVSMCs. Wound repair assays revealed that the time to closure was significantly affected by bFGF treatment. Cell coverage rate showed that, under untreated conditions, both visceral and subcutaneous tissues showed slower repair of the wounded area. However, treatment with bFGF for 9 days before T2DM ASCs were seeded into the chamber enhanced repair (*p*<0.05) to a similar extent as that found in non-T2DM ASCs (Fig. 3a).

Next, we used four chemotaxis assay set-ups to test potential paracrine effects of ASCs on HVSMCs. In all four set-ups, HVSMCs were seeded into the upper wells of transwell chambers. The lower wells contained various combinations of medium/cells/bFGF: (1) ASC medium (DMEM); (2) ASC medium (DMEM) plus 10 ng/ml bFGF; (3) non-T2DM ASCs; (4) subcutaneous T2DM ASCs; (5) subcutaneous T2DM ASCs treated for 9 days with bFGF; (6) visceral T2DM ASCs; and (7) visceral T2DM ASCs treated for 9 days with bFGF. After 4 h, ASCs treated with bFGF showed higher induction of chemotactic HVSMCs migration compared with untreated ASCs (Fig. 3b). The ASC culture medium alone was not able to induce HVSMC migration, even with addition of bFGF. This demonstrates that the chemotactic activity of ASCs was attributable to the cells themselves rather than the culture medium.

In addition, we performed co-culture experiments of T2DM ASCs and HVSMCs to further investigate the role of bFGF in T2DM ASC-induced recruitment of HVSMCs.T2DM ASCs previously treated with bFGF formed tube-like structures and HVSMCs localised around ASCs, providing support and stability to the capillary-like structures. Under basal conditions, the formation of tube-like structures was almost abrogated and HVSMCs were not able to migrate towards T2DM ASCs (Fig. 3c).

**Fig. 4:**
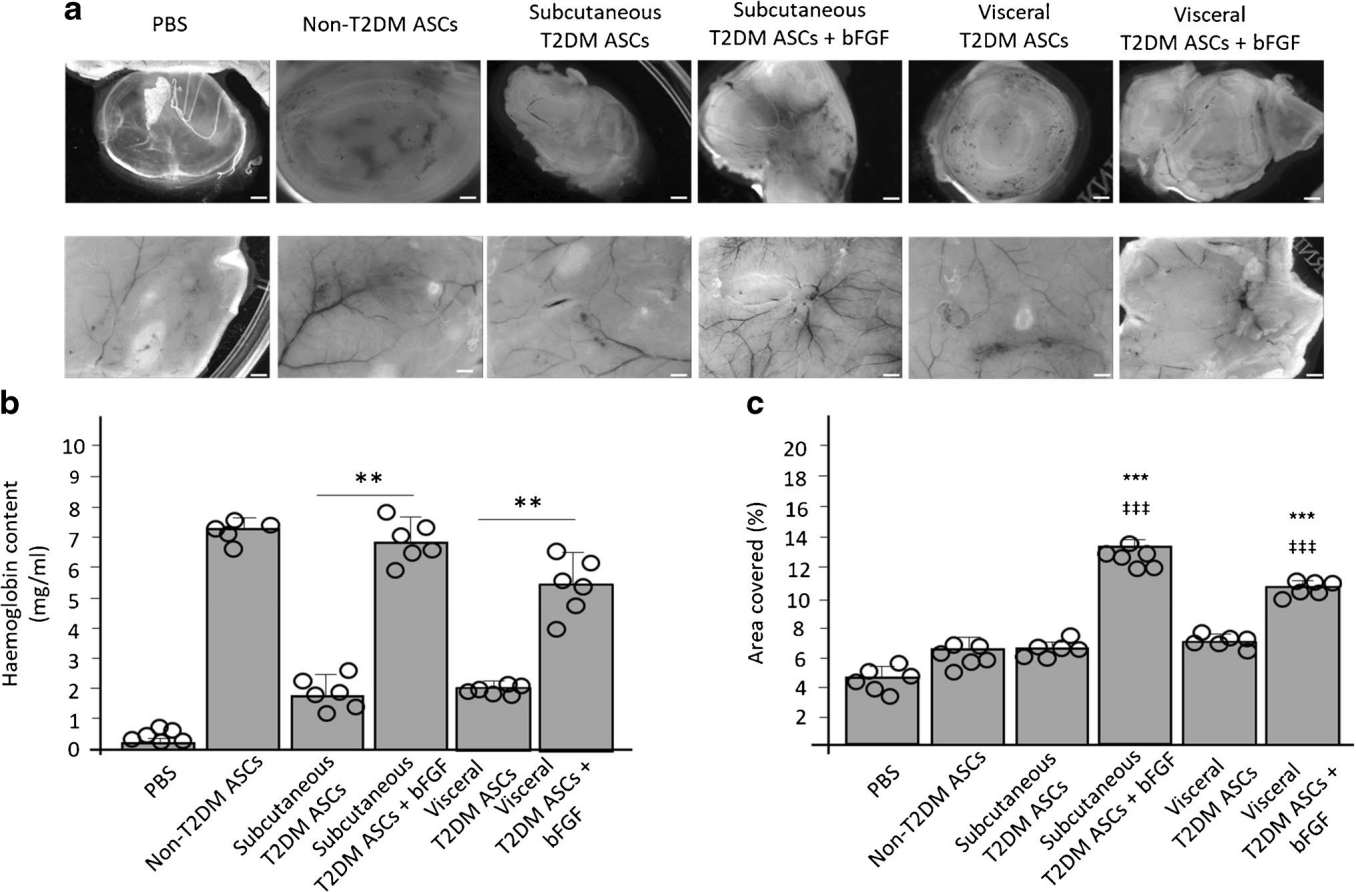
bFGF induces microvessel formation in Matrigel plugs in vivo. (**a**) Macroscopic views of Matrigel plugs (top) and underlying skin (bottom) 7 days after Matrigel plug injection. Plugs contain a co-culture of cells at a 1:1 ratio comprising non-T2DM ASCs and HVSMCs; subcutaneous T2DM ASCs and HVSMCs; subcutaneous T2DM ASCs previously treated for 9 days with bFGF and HVSMCs; visceral T2DM ASCs and HVSMCs; and visceral T2DM ASCs previously treated for 9 days with bFGF and HVSMCs. Scale bar, 1 mm. (**b**) Haemoglobin content in Matrigel implants measured by the cyanhaemoglobin method on day 7 and normalised to the weight of the analysed fragment. Values are means ± SD (*n*=6). Statistical analysis was performed by one-way ANOVA followed by Tukey test: **p<0.01, subcutaneous/visceral T2DM ASCs vs subcutaneous/visceral T2DM ASCs + bFGF. (**c**) Area covered by tubes. Values are means ± SD (*n*=6). Statistical analysis was performed by two-way ANOVA followed by Tukey test: ***p<0.001, non-T2DM ASCs and HVSMCs vs subcutaneous/visceral T2DM ASCs + bFGF and HVSMCs; ^‡‡‡^*p*<0.001, subcutaneous/visceral T2DM ASCs and HVSMCs vs subcutaneous/visceral T2DM ASCs + bFGF and HVSMCs

We also explored whether the crosstalk between subcutaneous and visceral T2DM ASCs and HVSMCs stimulates neovessel formation in vivo, by subcutaneously inoculating nude mice with Matrigel plugs. Co-cultures comprising PBS (control), subcutaneous non-T2DM ASCs + HVSMCs, subcutaneous T2DM ASCs treated with or without bFGF for 9 days + HVSMCs, and visceral T2DM ASCs treated with or without bFGF for 9 days + HVSMCs, were analysed. Macroscopic observation of the Matrigel plugs retrieved from mice at day 7 post-implantation showed that non-T2DM ASCs + HVSMCs and untreated subcutaneous and visceral T2DM ASCs + HVSMCs stimulated angiogenesis, resulting in formation of enlarged neovessels (Fig. 4a). Moreover, treatment of subcutaneous and visceral T2DM ASCs + HVSMCs with bFGF rescued the proangiogenic activity, as shown by the increased neovessel formation and haemoglobin content in Matrigel plugs (Fig. 4b, c).

## Discussion

ASCs present in adipose tissue are promising candidates for stem cell-based therapy. The mechanisms by which ASCs exert beneficial effects include multiple autocrine and paracrine signals [4, 5]. Nevertheless, it is known that ASCs obtained from individuals with type 2 diabetes are less effective in terms of proliferation, stemness, immunomodulation and angiogenesis [15]. This study analysed the transcriptomic profiles of subcutaneous and visceral ASCs from individuals with diabetes after bFGF treatment in culture. We demonstrate that bFGF regulates specific miRNAs involved in the cell proliferation and angiogenic capacity of ASCs, enhancing their potential as an autologous cell therapy.

In line with previous findings [29, 32], the proliferation capacity of T2DM ASCs was diminished compared with that of non-T2DM ASCs. Previous studies had explored the beneficial effects of bFGF in ASCs [33]. Our findings confirm that just 9 days of bFGF treatment significantly enhanced the proliferation rates of T2DM ASCs. Interestingly, bFGF not only enhanced the proliferation capacity of T2DM ASCs, but also affects their paracrine actions. We observed that bFGF-treated ASCs display a chemoattractant function towards HVSMCs, increasing their migratory capacity and accelerating wound closure. Moreover, co-culture with HVSMCs promotes the formation of enlarged neovessels, contributing to vascular remodelling.

miRNAs are involved in many cell mechanisms, such as proliferation, migration, stemness maintenance and differentiation of ASCs [21, 34–39], and their regulation may be modified by the presence of comorbidities such as obesity or diabetes mellitus [28, 29, 33]. It has been found that diabetes mellitus induces overexpression of miR-16, miR-24, miR-140, miR-145 and miR-146 and downregulation of miR-17, which suppress the ASC proliferation capacity [29]. Here, we observed that bFGF inhibited miR-140 and upregulated miR-17 expression, thus enhancing the viability and proliferation activity of T2DM ASCs. The gene ontology (GO) analysis also confirmed involvement of the predicted targeted genes in cell proliferation and migration.

The term angio-miR has emerged to identify specific miRNAs involved in the regulation of endothelial cell differentiation during the formation of new blood vessels network [40]. One of the main objectives in ASC studies is identification of specific miRNA signatures during maintenance, remodelling and mesenchymal differentiation, including vessel formation [1]. Of 19 angio-miRs inhibited after bFGF treatment, only three were common between subcutaneous and visceral T2DM ASCs (miR-143, miR-23b and miR-27b). Although it has been shown that subcutaneous and visceral T2DM ASCs have different miRNA transcriptomic profiles [13], we did not observe any differences in miRNA expression between subcutaneous/visceral non-T2DM ASCs and subcutaneous/visceral T2DM ASCs.

miR-17 is the only miRNA that has been identified as being regulated by bFGF, playing key roles in the proliferation and angiogenic properties of ASCs [29]. However, the effects of miR-17 have not yet been described. As a member of the miR-17–92 cluster, miR-17 is associated with various fundamental cellular functions, including proliferation, apoptosis and angiogenesis, through post-transcriptional regulation of target genes [41, 42]. Chamorro-Jorganes et al reported that inhibition of the miR-17–92 cluster decreased cumulative sprout length, the number of sprouts per spheroid and early branch point formation [42]. However, the regulation of miR-17 and miR-140 by bFGF had not yet been examined. In the present study, we demonstrate that bFGF upregulates miR-17 and inhibits miR-140 in T2DM ASCs. This regulation could stimulate the proliferation capacity and angiogenic properties of T2DM ASCs, improving cellular functions and promoting the formation, stabilisation and maturation of microvascular vessels. Culturing ASCs with bFGF appears to mitigate diabetes-related dysfunction by altering miRNA expression and modulating genes involved in vascular remodelling. These findings were further supported by community cluster analysis, which identified two clusters related to vascular remodelling and another cluster related to endothelial cell biology.

In conclusion, bFGF regulates the miRNA profile of subcutaneous and visceral T2DM ASCs, restores stem cell function impaired by diabetes, and promotes the formation, stabilisation and maturation of microvascular vessels, thereby enhancing the angiogenic properties of ASCs. Limitations of this study include the limited number of samples due to the difficulty in obtaining both subcutaneous and visceral samples from the same individuals with type 2 diabetes, and in expanding these samples, as well as the different age ranges between individuals with or without type 2 diabetes.

## Data Availability

The datasets used and/or analysed during the current study are available from the corresponding author on reasonable request. The GSE245003 miRNA profile was downloaded from the Gene Expression Omnibus (GEO; https://www.ncbi.nlm.nih.gov/geo/). The new data discussed in this publication have been deposited in GEO and are accessible through GEO accession number GSE283040.

## Abbreviations

ASC: Adipose-derived stem cell
bFGF: Basic fibroblast growth factor
FC: Fold change
HVSMCs: Human vascular smooth muscle cells
mirDIP: miRNA data integration protocol
Non-T2DM ASCs: ASCs from individuals without type 2 diabetes
T2DM ASCs: ASCs from individuals with type 2 diabetes
